# Community music activity participation and loneliness among Chinese young adults living alone

**DOI:** 10.3389/fpubh.2026.1930459

**Published:** 2026-09-03

**Authors:** Yundi Pian, Yafei Yu

**Affiliations:** 1 School of Music, Nanjing Normal University, Nanjing, China; 2 Department of Musicology, Xinghai Conservatory of Music, Guangzhou, China

**Keywords:** community music activity participation, loneliness, meaning in life, perceived restorativeness, structural equation modeling (SEM), young adults living alone

## Abstract

**Background:**

Loneliness has become an increasingly salient psychological and social issue among young adults in contemporary urban China. Young adults living alone may be particularly vulnerable because of reduced daily companionship, limited opportunities for sustained social interaction, and relatively weak emotional support networks. As an accessible form of cultural and social participation embedded in community settings, community music activity may be relevant to loneliness among young adults living alone. However, the associations linking community music participation, perceived restorativeness, meaning in life, and loneliness among Chinese young adults living alone remain underexplored.

**Methods:**

This cross-sectional study included 981 Chinese young adults living alone who had prior experience of community music activities. Structural equation modeling (SEM) was used to examine the association between community music activity participation and loneliness, together with indirect associations involving perceived restorativeness and meaning in life.

**Results:**

Among young adults living alone with prior community music participation, higher levels of community music activity participation were associated with lower loneliness. Perceived restorativeness and meaning in life were each involved in significant indirect associations between variation in community music participation and loneliness, and a serial indirect association involving perceived restorativeness and meaning in life was also observed. The standardized serial indirect association was relatively modest (−0.023) compared with the standardized total association (−0.384), while the standardized direct association remained −0.200. These effect estimates indicate that the proposed indirect pathways provide a partial rather than comprehensive statistical explanation of the association between variation in community music participation and loneliness within this participant sample.

**Conclusion:**

Among Chinese young adults living alone who had prior experience of community music participation, higher participation was associated with lower loneliness, with perceived restorativeness and meaning in life involved in the observed indirect associations. The findings provide preliminary evidence that community music participation may be experienced in ways consistent with ART-related restorative dimensions and may offer a meaningful social and psychological context relevant to loneliness. However, given the cross-sectional design and the absence of an active non-musical community-participation comparison group, the findings do not establish causal processes, validate an extension of ART to community music, or demonstrate that these experiences are unique to music. Community music should therefore be regarded as a promising context for future longitudinal and intervention research rather than an established loneliness-reduction intervention.

## 1 Introduction

In recent years, loneliness has become an increasingly important topic in social science and mental health research (1). As urbanization, population mobility, and changes in living arrangements continue to reshape everyday social life, individuals may have greater lifestyle flexibility without necessarily gaining stable and sustained emotional connections (2, 3). Loneliness is therefore increasingly understood as a subjective social and psychological experience related to the perceived quality of interpersonal relationships and social connectedness (4). Within this broader context, young adults living alone have attracted increasing attention in urban China (5). Educational mobility, employment-related relocation, and changing housing arrangements have contributed to a growing number of young adults living independently for extended periods (6). For some individuals in this group, reduced daily companionship, fewer opportunities for sustained interpersonal interaction, and relatively limited emotional support may be associated with stronger feelings of loneliness (7). Their experiences of loneliness are therefore embedded not only in individual psychological states but also in everyday living environments, social relationships, and opportunities for social participation (8, 9).

Community settings provide one relevant context in which these social experiences may be examined. Because communities are closely connected to housing, leisure, and neighborhood interaction, community-based activities can provide opportunities for repeated interpersonal contact and shared participation (10–12). For young adults living alone, such accessible and recurring forms of participation may be relevant to perceptions of social connection and belonging (13). Community music represents one form of socially engaging community participation. Activities such as collective singing, instrumental performance, rehearsal, and shared listening commonly involve shared presence, coordination, interpersonal interaction, and emotional engagement (14–16). These characteristics may provide opportunities for social and emotional connection. However, similar qualities may also be present in other forms of collective community participation, including group exercise, volunteering, community arts, and sports. Community music should therefore be understood as one specific participatory context in which socially meaningful experiences may occur, rather than as an activity whose effects can be assumed to be unique to music.

Existing studies have linked music and community music participation with social connectedness, belonging, emotional well-being, and other positive social experiences (17–19). However, relatively limited attention has been paid specifically to Chinese young adults living alone, and the psychological correlates involved in the association between community music participation and loneliness remain insufficiently examined (20). One potentially relevant construct is perceived restorativeness. Originating primarily from restorative-environment research, perceived restorativeness refers to individuals’ evaluations of whether an environment or experiential context possesses qualities associated with psychological restoration. Attention Restoration Theory (ART) identifies being away, fascination, extent, and compatibility as central restorative dimensions. Although these concepts have traditionally been examined mainly in relation to natural and physical environments, subsequent research has considered restorative qualities in auditory, multisensory, and other experiential contexts (21–26).

From this perspective, community music participation may be examined as an active and socially embedded experiential context in which participants perceive qualities conceptually consistent with ART. Musical engagement may provide temporary psychological distance from everyday pressures; rhythm, melody, and collective involvement may be associated with fascination; rehearsal and coordinated performance may provide experiential continuity and structure; and participants may perceive compatibility between their needs, modes of participation, and the social–emotional atmosphere of the activity. Importantly, applying these ART-related dimensions to community music does not imply that community music is equivalent to a natural restorative environment or that ART has already been empirically validated as extending to active music participation. Rather, perceived restorativeness provides a theoretical framework for examining whether participants evaluate community music experiences in ways consistent with restorative dimensions.

Meaning in life provides a second psychological perspective relevant to the present study. Community music participation may involve opportunities for social connection, self-expression, coordinated engagement, and a sense of competence, all of which may be associated with meaning-related evaluations. Self-Determination Theory offers a complementary interpretive framework by emphasizing the relevance of autonomy, competence, and relatedness to positive psychological functioning. In the present study, ART is used primarily to interpret perceived restorativeness, whereas Self-Determination Theory provides a complementary perspective for considering meaning-related experiences. These perspectives are therefore used to organize theoretically related associations rather than to establish a confirmed temporal or causal psychological process. The present study does not empirically test an integrated ART–SDT model because autonomy, competence, and relatedness were not directly measured.

Against this background, the present study examines the associations among community music activity participation, perceived restorativeness, meaning in life, and loneliness in a sample of 981 Chinese young adults living alone with prior community music participation. Perceived restorativeness and meaning in life are incorporated into a theoretically specified serial indirect-association model to examine whether the observed data are consistent with the proposed relationships among these constructs. Because all respondents had prior experience of community music activities, the study concerns variation in participation experiences within this participant sample rather than differences between participants and non-participants. Moreover, because no active non-musical community-participation comparison group was included, the study cannot determine whether the observed restorative, relational, or meaning-related associations are unique to musical engagement. Given the cross-sectional design, the proposed model should be interpreted as a representation of concurrent associations rather than as evidence of a causal or temporally established sequence.

The study therefore addresses three related gaps. First, it focuses on young adults living alone in the Chinese urban context, a population for whom community participation may be particularly relevant to experiences of social connection and loneliness. Second, it examines perceived restorativeness and meaning in life as psychological correlates involved in the association between community music participation and loneliness. Third, it explores the theoretical applicability of ART-related restorative dimensions within an active, socially immersive, and culturally embedded participatory context, while treating this application as preliminary rather than as definitive empirical validation of an extension of ART.

## 2 Literature review and research hypotheses

### 2.1 Participation in community music activities and loneliness

Participation in community music activities represents an extension of music participation research into community settings, emphasizing how individuals establish connections with others, their communities, and their cultural environments through musical practice (27). Stige (28) further points out that engagement in musical activities is not merely an individual artistic behavior but also a collaborative and communal practice that emphasizes human interaction and shared experiences within music. Existing studies generally suggest that participation in musical activities may contribute to emotional well-being and social connectedness by facilitating interpersonal interaction and shared experiences. For example, Hallam et al. (29) found that participation in musical activities can positively influence emotional, cognitive, and social functioning, including enhancing a sense of autonomy and social belonging. Building on this perspective, some scholars have increasingly incorporated the concept of “community” into research on musical participation. English and Corderoy (30) found that participation in community music activities can help participants build interpersonal connections, strengthen their sense of belonging, and foster motivation for continued participation. Similarly, Vougioukalou et al. (31) argued that community music activities provide opportunities for individuals from diverse cultural backgrounds to express themselves and communicate, thereby promoting social integration and cultural understanding. Heard and Bartleet (18) further suggested that community music participation may enhance not only individual well-being but also self-expression, participation, and collective identity formation. Taken together, these studies indicate that participation in community music activities involves both individual developmental and social relational dimensions. In addition to facilitating musical engagement, community music participation may strengthen interpersonal connectedness, cultural exchange, and collective identity within community contexts. Based on this, the present study defines participation in community music activities as the process through which individuals establish social connections and engage in cultural interaction through musical practices such as singing, instrument playing, and composing within specific community contexts, thereby fostering a sense of belonging and shared meaning.

Loneliness refers to a subjective negative experience that arises when individuals perceive that their existing social relationships are unable to meet their emotional or social needs (32). Previous research has emphasized that loneliness is not equivalent to objective social isolation; rather, it reflects an individual’s perceived deficiency in meaningful social connection and the psychological distress arising from discrepancies between actual and desired relationships (33). Existing studies generally suggest that loneliness is closely associated with both psychological well-being and broader patterns of social interaction. Building on this foundation, recent research on loneliness has mainly focused on three aspects. First, studies have extensively examined the effects of loneliness on physical and mental health. For example, Lim et al. (34) highlighted the association between loneliness and psychological problems such as depression, anxiety, and cognitive decline, suggesting that loneliness is increasingly recognized as an important public health issue. Second, increasing attention has been paid to loneliness among different demographic groups, particularly young people. Alam et al. (35) noted that recent studies on youth loneliness have focused on its relationship with persistent psychological difficulties and emphasized the importance of distinguishing transient loneliness from chronic loneliness. Finally, with the development of digital society, research has increasingly explored intervention approaches for loneliness, particularly the potential role of digital technologies and remote support systems. However, existing evidence also suggests that the long-term effectiveness and applicability of such interventions remain inconclusive (36). Taken together, current studies indicate that loneliness research has gradually expanded from conceptual discussions to broader concerns involving health consequences, demographic differences, and intervention strategies. However, relatively limited attention has been paid to loneliness experiences among young adults living alone within specific sociocultural contexts. This provides an important basis for the present study to further examine loneliness among young adults living alone in China.

**Figure 1:**
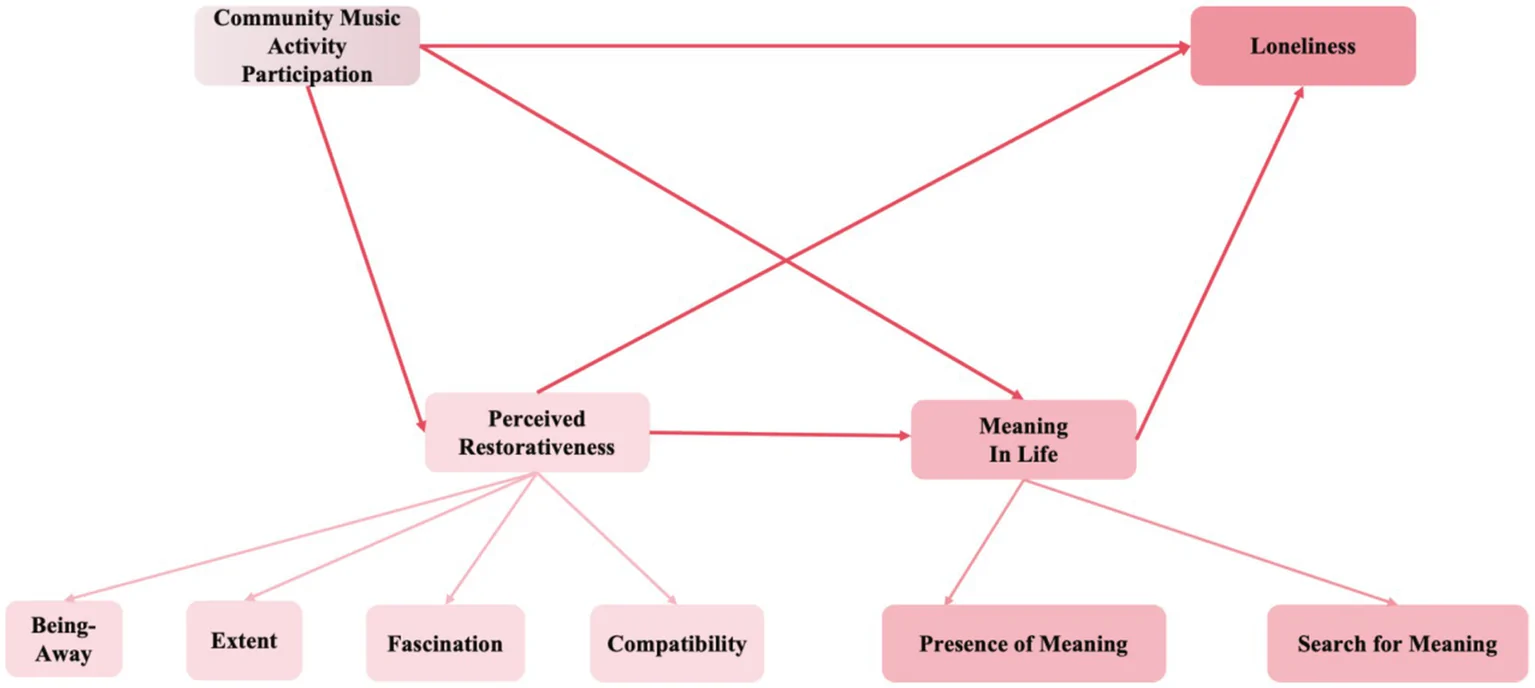
Proposed conceptual model.

In a community context, the relationship between participation in community music activities and feelings of loneliness can be understood through the lens of “social bond formation.” Tierney et al. (37) noted that community-based activities can promote the accumulation of social capital through the establishment of trust, group cohesion, and reciprocal relationships, thereby enhancing individuals’ sense of belonging and fostering meaningful interpersonal connections. This suggests that the significance of community participation lies not merely in the activities themselves, but also in their capacity to facilitate sustained social relationships and emotional connectedness within communities. Similarly, Moore et al. (38) found that feelings of loneliness among young people are closely associated with low levels of community interaction, limited shared activities, and a weak sense of belonging, whereas shared interests, common goals, and stable social relationships may enhance social connectedness. These findings indicate that loneliness among young people may be shaped not only by individual psychological factors but also by the quality of social relationships embedded within community settings. Building on this perspective, Rickard et al. (39) suggested that musical activities may strengthen social connections among participants by fostering shared experiences, common values, and an ongoing sense of community, and are therefore increasingly viewed as a promising community-level approach for alleviating loneliness. Pearce et al. (40) further argued that social strategies involving continuous social support, opportunities for peer interaction, and shared activity environments tend to be more effective in reducing feelings of loneliness among young people. Taken together, existing studies suggest that participation in community music activities may be an important contextual factor associated with community-based social interaction and lower loneliness among young people. Through shared musical engagement and sustained interpersonal interaction, community music participation may be associated with stronger social connectedness and lower feelings of loneliness. Based on this, this paper proposes the following hypothesis. Figure 1 illustrates the proposed conceptual framework of this study, including the relationships among community music participation, perceived restorativeness, meaning in life, and loneliness.

*H1:* Community music activity participation is negatively associated with loneliness among young adults living alone who had participated in community music activities.

### 2.2 The indirect role of perceived restorativeness

Perceived restorativeness primarily originates from research on restorative environments and generally refers to an individual’s subjective perception of whether a particular environment or situation possesses the capacity to restore psychological resources, alleviate stress, and promote physical and mental recovery (21). Han (22) further notes that the concept emphasizes individuals’ subjective evaluations of an environment’s restorative potential, particularly whether the environment enables recovery from daily exhaustion, attentional fatigue, or psychological stress. It is therefore necessary to clarify what is meant by “environment” in the present study. In early ART research, the environment was often understood as a physical environment, such as natural landscapes, parks, or other material spaces. In the present study, however, community music activities are conceptualized as a psychological and experiential environment. This type of environment is not defined solely by physical features, but is jointly produced through musical activity, interpersonal interaction, emotional resonance, shared rhythm, and sensory immersion. This distinction is important because young adults living alone may experience restoration not only by entering a particular place, but also by participating in a structured and affectively meaningful activity context.

Attention Restoration Theory (ART), proposed by Kaplan, explains how individuals restore attentional and psychological resources after prolonged directed attention. According to ART, restorative environments generally possess four core characteristics: “being away,” “fascination,” “extent,” and “compatibility (23, 24).” These characteristics provide the theoretical bridge for applying perceived restorativeness to community music activities. Specifically, community music activities may create a sense of “being away” by allowing participants to temporarily detach from work pressure, solitary routines, and repetitive daily concerns. They may evoke “fascination” through rhythm, melody, embodied listening, and immersive auditory engagement, which can attract attention without excessive cognitive effort. They may provide “extent” because rehearsals, collective singing, instrumental coordination, and shared musical processes create a coherent experiential field with continuity and internal structure. They may also foster “compatibility” when the emotional atmosphere, participatory rules, and social expectations of the activity fit participants’ needs for relaxation, expression, and interpersonal connection. In this sense, the restorative potential of community music activities does not rely only on the physical venue in which the activity takes place, but on the functional restorative qualities associated with participation itself.

Previous research extending restorative-environment research to auditory and soundscape contexts suggests that restorative evaluations are not limited to visual-natural settings (25, 26), providing a conceptual basis for examining perceived restorativeness in active community music contexts. With regard to loneliness, young people’s loneliness experiences are closely associated with their subjective perceptions of social contexts and relational connectedness (41). Therefore, if community music participation is associated with a restorative psychological space characterized by emotional soothing, shared attention, and interpersonal resonance, this perceived restorativeness may be further related to participants’ evaluations of the activity context and social relationships, thereby being associated with lower levels of loneliness. Based on this, this paper proposes the following hypothesis:

*H2:* Perceived restorativeness is involved in an indirect association between community music activity participation and loneliness among young adults living alone with prior community music participation.

### 2.3 The indirect role of meaning in life

Meaning in life generally refers to individuals’ perceptions of purpose, value, coherence, and significance in their lives. It includes two conceptually distinct dimensions, presence of meaning (PM) and search for meaning (SM), which reflect related aspects of meaning-related experience (42). In the present study, PM and SM are treated as two related dimensions of a broader meaning-in-life construct rather than as separate predictors. Their moderate latent correlation (*r* = 0.491) suggests that the two dimensions are related but not redundant.

Previous research has identified meaning in life as a psychological resource associated with mental health, identity development, self-understanding, and supportive interpersonal experiences (43–46). Self-Determination Theory (SDT) provides a complementary framework for interpreting these associations by emphasizing autonomy, competence, and relatedness as important psychological needs (47, 48). In community music contexts, shared participation, self-expression, coordinated engagement, and interpersonal interaction may be associated with experiences of competence, connectedness, autonomy, and personal growth (49, 50). These findings provide a theoretical basis for examining meaning in life as a psychological correlate of community music participation.

Meaning in life has also been associated with loneliness. Macià et al. (51) reported that stronger meaning in life was associated with lower loneliness, while Folker et al. (52) highlighted its relevance to relational connectedness and coping with loneliness. Accordingly, among young adults living alone with prior community music participation, variation in participation may be associated with meaning-related experiences, which may in turn be associated with loneliness. Based on this, the following hypothesis is proposed:

*H3:* Meaning in life is involved in an indirect association between community music activity participation and loneliness among young adults living alone with prior community music participation.

### 2.4 Proposed serial indirect association

Existing research provides a theoretical basis for considering perceived restorativeness and meaning in life jointly in relation to community music participation and loneliness. A review by English et al. (53) indicated that positive experiences associated with community music participation involve not only self-perception and quality of life, but also personal growth and transformative experiences emerging within musical activities. Gomes et al. (17) similarly reported that community music participation is associated with trust, empathy, mutual support, belongingness, and personal fulfillment. Together, these findings suggest that community music participation may be associated with psychologically supportive and socially inclusive activity contexts in which participants experience both relational engagement and emotional restoration.

Perceived restorativeness and meaning in life may represent two conceptually related but distinct aspects of these experiences. Restorative evaluations concern how an activity context is perceived as providing psychological distance from everyday pressures, attentional engagement, and experiential compatibility. Lundvall et al. (54), for example, noted that well-being among young people is associated with experiences of finding a psychologically supportive “haven” in which they can distance themselves from everyday pressures and regain a sense of stability. Meaning in life, by contrast, concerns broader evaluations of purpose, value, and significance. Previous research has shown that a stronger sense of community is associated with both lower loneliness and greater perceived meaning in life (55), while a large-scale meta-analysis reported that stronger life purpose is associated with lower concurrent loneliness and a reduced risk of future loneliness (56). These findings provide a theoretical basis for examining whether restorative and meaning-related evaluations are jointly involved in the association between community music participation and loneliness.

Accordingly, the present study specifies a serial indirect association in which community music participation is associated with perceived restorativeness, perceived restorativeness is associated with meaning in life, and meaning in life is associated with loneliness. This ordering reflects the proposed theoretical structure of the model rather than an established temporal sequence. Given the cross-sectional design, the present study cannot determine whether restorative experiences occur before changes in meaning in life or whether either construct subsequently produces changes in loneliness. The proposed model therefore tests whether the observed data are consistent with this theoretically specified pattern of associations.

*H4:* Perceived restorativeness and meaning in life are involved in a serial indirect association between community music activity participation and loneliness among young adults living alone with prior community music participation.

## 3 Methods

### 3.1 Study design and participants

This cross-sectional study examined the associations between community music activity participation and loneliness among Chinese young adults living alone with prior experience of community music activities. Participants were recruited from community music activity settings in three first-tier Chinese cities (Shanghai, Beijing, and Shenzhen).

Eligible participants were required to meet the following criteria: (1) aged 18–40 years; (2) currently living alone for at least six consecutive months; and (3) having participated in at least one community music activity within the previous year, including community singing, instrumental ensemble activities, music workshops, rehearsals, or other collective music events organized in community settings. In this study, young adults living alone were operationally defined as individuals aged 18–40 years who had maintained an independent living arrangement for at least 6 months.

Participants were recruited through cooperation with community staff and activity organizers. Paper-based questionnaires were distributed and collected on site before or after community music activities. Before completing the questionnaire, participants were informed about the study objectives, voluntary participation, confidentiality, and their right to withdraw. Written informed consent was obtained from all participants.

The study protocol was reviewed and approved by the Institutional Review Board of Xinghai Conservatory of Music (Approval No.: RCTXCM20251201). The study was conducted in accordance with the principles of the Declaration of Helsinki.

No formal *a priori* sample size calculation was conducted before data collection. The final sample size was determined by the number of eligible participants who could be recruited during the study period across the selected community music activity settings. After data screening and quality control, 981 valid questionnaires were retained for the final analysis. A post-hoc power analysis conducted using G-Power 3.1 indicated that this sample size provided adequate statistical power to detect the hypothesized associations.

### 3.2 Measures

In this study, all study variables were assessed using established instruments. Except for loneliness, which was measured using a 4-point response format, all scales used a 5-point Likert scale ranging from 1 (strongly disagree) to 5 (strongly agree).

The Group Music Activity Participation (GMP) Scale adopted in this study was developed by Yang et al. (16) and consists of 4 items. The scale measures participants’ behavioral engagement in group music activities, including participation frequency, effortful involvement, cooperative behavior, and perceived skill and social development. We adapted this scale from the school context to the community context by replacing the contextual keyword “school” with “community,” while retaining all four original items, the response format, and the scoring procedure. This adaptation logic is consistent with the original scale developers’ approach.

*Regarding the measurement of perceived restorativeness (PR)*: this study adopted the Chinese version of the Perceived Restorativeness Scale revised by Li et al. (57) and contextually adapted its items to reflect the setting of community music activities. The scale comprises four dimensions: being away (BE), extent (EX), fascination (FA), and compatibility (CO). Examples of the adapted items include: “Participating in community music activities allows me to temporarily set aside everyday stress” (BE), “The rhythms and interactions in community music activities naturally draw my attention” (FA), “Community music activities provide me with a rich and immersive experiential world” (EX), and “During community music activities, I feel that my actions and the atmosphere are well matched” (CO). The adaptation preserved the core conceptual meaning of each dimension while tailoring the wording to the community music activity context. The contextual adaptation followed a systematic procedure. Three psychology professors reviewed the original items, and references to natural or physical environments were replaced with music-activity-specific contexts while preserving the conceptual meaning of each ART dimension. No new items or dimensions were introduced during the adaptation. The Cronbach’s α coefficients for BE, EX, FA, and CO in the present study were 0.840, 0.858, 0.890, and 0.868, respectively. In other words, the scale measures participants’ subjective restorative perceptions of their community music participation experience.

*Regarding the measurement of meaning in life (ML)*: this study utilized the Chinese version of the Meaning in Life Questionnaire (MLQ) revised by Liu and Gan (58). Due to ambiguities in the original translation, this version omits Item 10 from the original questionnaire and consists of nine items divided into two dimensions: presence of meaning (PM) and search for meaning (SM). Typical items include “I am searching for something that makes my life feel meaningful” and “I understand the meaning of my life.” The Cronbach’s α coefficients for PM and SM in the present study were 0.902 and 0.840, respectively.

*Regarding the measurement of loneliness (LO)*: this study employed a shortened version of the UCLA Loneliness Scale (ULS) for measurement. The UCLA Loneliness Scale is one of the most commonly used tools for measuring an individual’s subjective sense of loneliness, and several abbreviated versions have since been developed, including the 10-item version (ULS-10), the 8-item version (ULS-8), and the 6-item version (ULS-6). After comparing the suitability of each version, this study ultimately selected the Chinese translation of the ULS-6 (59). It is widely used in China and has been validated by numerous Chinese scholars, demonstrating good reliability and validity among the Chinese adult population. It is suitable for assessing the level of loneliness among Chinese adults. The ULS-6 consists of 6 items and uses a 4-point rating scale (60). Typical items include “I feel a lack of companionship” and “I feel I have little in common with others.” Cronbach’s alpha coefficient is 0.920.

Sociodemographic information was collected using a structured questionnaire. Participants reported their gender, age, marital status, education level, employment status, housing arrangement, and frequency of community music activity participation.

Gender was assessed as male or female. Age was recorded in years and categorized in Table 1 for descriptive purposes, while treated as a continuous variable in the structural model. Marital status was assessed as unmarried, married, or divorced/other. Education level was categorized as high school or below, junior college, bachelor’s degree, and master’s degree or above. Employment status was categorized as student, full-time employee, freelancer/self-employed, and unemployed/other. Housing arrangement was assessed as renting alone, self-owned housing, or other arrangements. Frequency of community music activity participation was categorized as 1–2 times, 3–5 times, 6–10 times, and more than 10 times in the previous year.

**Table 1:** Demographic characteristics of the participants (*N* = 981).

Variable	Category	*N*	%
Gender	Male	428	43.6
	Female	553	56.4
Age	18–21 years	96	9.8
	22–25 years	318	32.4
	26–30 years	356	36.3
	31–35 years	167	17.0
	36–40 years	44	4.5
Marital status	Unmarried	742	75.6
	Married	186	19.0
	Divorced / Other	53	5.4
Education	High school or below	72	7.3
	Junior college	214	21.8
	Bachelor’s degree	531	54.1
	Master’s degree or above	164	16.7
Employment status	Student	148	15.1
	Full-time employee	621	63.3
	Freelancer / Self-employed	127	12.9
	Unemployed / Other	85	8.7
Housing arrangement	Renting alone	603	61.5
	Living alone in self-owned housing	214	21.8
	Other	164	16.7
Frequency of community music activity participation in the past year	1–2 times	274	27.9
	3–5 times	356	36.3
	6–10 times	221	22.5
	More than 10 times	130	13.3

### 3.3 Data collection and quality control

Data collection was conducted from December 2025 to February 2026. Before the formal survey, a pilot study was conducted among 32 young adults living alone recruited from community music activity settings in Shenzhen. The pilot study evaluated item clarity, readability, and contextual appropriateness of the adapted questionnaire. Minor wording adjustments were made based on participant feedback and expert review, without changing the original construct structure or scale dimensions.

During the formal survey, participants completed paper-based questionnaires independently on site before or after relevant community music activities, and completed questionnaires were collected immediately after completion. A total of 1,100 questionnaires were distributed, and 1,028 questionnaires were returned. Quality-control procedures were conducted to ensure data validity and included screening for missing responses, inconsistent eligibility information, obvious response patterns, and other invalid cases. After excluding 47 questionnaires that were considered invalid based on these criteria, 981 valid questionnaires were retained for the final analysis.

### 3.4 Statistical analysis

Structural equation modeling (SEM) was conducted using AMOS 24.0 with maximum likelihood estimation. The measurement model was first evaluated using confirmatory factor analysis (CFA). Model fit was assessed using *χ*^2^/df, RMSEA, SRMR, GFI, CFI, TLI, and NFI. Acceptable model fit was indicated by *χ*^2^/df < 3, RMSEA and SRMR < 0.08, and incremental fit indices above 0.90.

The structural model examined the direct association between community music activity participation and loneliness, as well as indirect associations involving perceived restorativeness and meaning in life. Loneliness was modeled using the composite score of the ULS-6 because of its established unidimensional structure and high internal consistency in the present sample.

Gender was coded as 0 = male and 1 = female. Age was treated as a continuous variable. Marital status was dummy-coded with unmarried participants as the reference category. Education level was treated as an ordinal variable (1 = high school or below, 2 = junior college, 3 = bachelor’s degree, 4 = master’s degree or above). Employment status was dummy-coded with full-time employment as the reference category. Housing arrangement was dummy-coded with renting alone as the reference category. Frequency of community music activity participation was treated as an ordinal continuous variable (1 = 1–2 times, 2 = 3–5 times, 3 = 6–10 times, 4 = more than 10 times).

Indirect associations were tested using bias-corrected bootstrap estimation with 5,000 resamples. Statistical significance was determined when the 95% bootstrap confidence interval did not include zero.

Missing data were examined before analysis. Because missing responses accounted for less than 3% of all items and Little’s MCAR test indicated that missingness was completely random (*p* = 0.31), missing values were handled using the expectation–maximization algorithm.

Sensitivity analyses were conducted to examine the robustness of the findings, including alternative model specifications and additional robustness checks.

## 4 Results

### 4.1 Participant characteristics

A total of 981 valid questionnaires were included in the final analysis. Table 1 presents the demographic characteristics of the participants. The sample included 428 males (43.6%) and 553 females (56.4%). Most participants were aged between 22 and 30 years (68.7%), and the majority were unmarried (75.6%). Regarding educational background, most participants had received college-level education or above, with bachelor’s degree holders representing the largest proportion (54.1%). In terms of employment status, 63.3% of participants were full-time employees, followed by students (15.1%) and freelancers/self-employed individuals (12.9%). Most participants lived alone in rented housing (61.5%). Regarding community music activity participation, 27.9% reported participating 1–2 times in the previous year, 36.3% reported 3–5 times, 22.5% reported 6–10 times, and 13.3% reported more than 10 times.

### 4.2 Measurement model assessment and common method bias

Confirmatory factor analysis (CFA) was conducted to evaluate the measurement model. The measurement model demonstrated satisfactory fit: *χ*^2^/df = 2.253, RMSEA = 0.036, SRMR = 0.026, GFI = 0.973, NFI = 0.953, TLI = 0.969, and CFI = 0.973.

All standardized factor loadings exceeded 0.70. The average variance extracted (AVE) values ranged from 0.569 to 0.768, and composite reliability (CR) values ranged from 0.840 to 0.920, indicating satisfactory convergent validity and internal consistency.

The four dimensions of perceived restorativeness (being away, extent, fascination, and compatibility) showed moderate correlations, supporting their conceptual representation as dimensions of a higher-order construct. Discriminant validity was examined using the Fornell–Larcker criterion and the heterotrait–monotrait ratio (HTMT). The square roots of AVE values exceeded correlations with other constructs, and all HTMT values were below 0.85, supporting satisfactory discriminant validity. Detailed CFA results and validity statistics are provided in Supplementary material (Appendix Tables A1, A2).

Common method bias was assessed using Harman’s single-factor test and confirmatory factor comparison. The first unrotated factor accounted for 28.75% of total variance, below the commonly used 40% threshold. In addition, the single-factor model showed substantially poorer fit than the measurement model (single-factor model: *χ*^2^/df = 28.30, RMSEA = 0.167, SRMR = 0.147, GFI = 0.45, CFI = 0.37). These results indicated that severe common method bias was unlikely.

### 4.3 Descriptive statistics and correlations

Pearson correlation analyses were conducted to examine associations among the main variables (Table 2). Community music activity participation was positively correlated with perceived restorativeness (*r* = 0.338, *p* < 0.01) and meaning in life (*r* = 0.461, *p* < 0.01), and negatively correlated with loneliness (*r* = −0.351, *p* < 0.01). Perceived restorativeness and its dimensions were positively associated with meaning in life and negatively associated with loneliness. Meaning in life was negatively correlated with loneliness (*r* = −0.299, *p* < 0.01). All major study variables showed significant correlations in the expected directions.

**Table 2:** Correlation analysis results for key variables.

Variable	CMAP	BE	EX	FA	CO	PM	SM	LO	PR	ML
CMAP	1									
BE	0.241**	1								
EX	0.281**	0.440**	1							
FA	0.231**	0.474**	0.470**	1						
CO	0.271**	0.418**	0.433**	0.361**	1					
PM	0.348**	0.180**	0.219**	0.170**	0.182**	1				
SM	0.433**	0.247**	0.254**	0.256**	0.221**	0.431**	1			
LO	−0.351**	−0.204**	−0.215**	−0.257**	−0.179**	−0.242**	−0.265**	1		
PR	0.338**	0.761**	0.771**	0.762**	0.737**	0.248**	0.323**	−0.282**	1	
ML	0.461**	0.252**	0.280**	0.252**	0.238**	0.850**	0.842**	−0.299**	0.337**	1

**p < 0.01.

### 4.4 Structural model and direct/indirect associations

**Figure 2:**
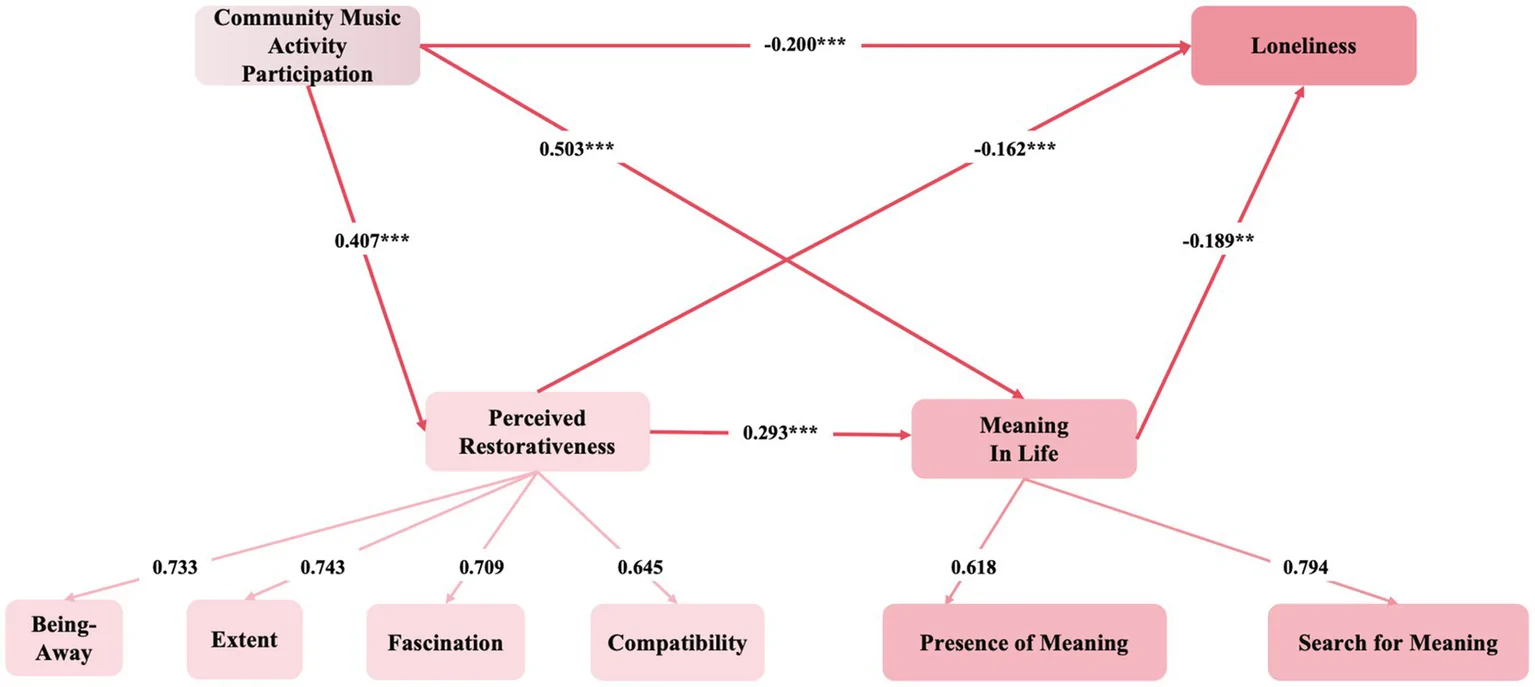
Diagram of the revised structural equation model. ^***^p < 0.001, ^**^p < 0.01.

Figure 2 presents the structural equation model tested in this study. The model exhibited an acceptable overall fit to the data: *χ*^2^ = 877.049, df = 393, *χ*^2^/df = 2.232 (< 3). Additional fit indices all met the recommended thresholds: GFI = 0.943 (> 0.9), AGFI = 0.932 (> 0.9), RMSEA = 0.035 (< 0.08), CFI = 0.973 (> 0.9), NFI = 0.951 (> 0.9), RFI = 0.946 (> 0.9), TLI = 0.970 (> 0.9), and IFI = 0.973 (> 0.9). These results indicate that the proposed model fits the observed data well and is suitable for subsequent path analysis.

As reported in Table 3, community music activity participation (CMAP) was positively associated with perceived restorativeness (PR) (*β* = 0.407, *p* < 0.001) and meaning in life (ML) (*β* = 0.503, *p* < 0.001). Perceived restorativeness was also positively associated with meaning in life (*β* = 0.293, *p* < 0.001). In addition, the maximum-likelihood estimates showed that meaning in life (standardized *β* = −0.189, *p* = 0.003), community music activity participation (standardized *β* = −0.200, *p* < 0.001), and perceived restorativeness (standardized *β* = −0.162, *p* < 0.001) were negatively associated with loneliness. These results supported the hypothesized associations among the core latent variables.

**Table 3:** Path coefficients in the structural equation model.

Path	Unstandardized coefficient	S.E.	C.R.	ML-based p	Standardized coefficient
CMAP → PR	0.265	0.027	9.813	< 0.001	0.407
CMAP → ML	0.300	0.031	9.598	< 0.001	0.503
PR → ML	0.268	0.046	5.784	< 0.001	0.293
ML → LO	−0.233	0.078	−2.976	0.003	−0.189
CMAP → LO	−0.147	0.036	−4.057	< 0.001	−0.200
PR → LO	−0.183	0.051	−3.564	< 0.001	−0.162
ML → PM	1.000				0.618
ML → SM	1.109	0.099	11.166	< 0.001	0.794
PR → CO	1.003	0.074	13.596	< 0.001	0.645
PR → FA	1.117	0.077	14.476	< 0.001	0.709
PR → EX	1.067	0.074	14.414	< 0.001	0.743
PR → BE	1.000				0.733
CMAP → CMAP1	1.000				0.857
CMAP → CMAP2	0.956	0.030	31.648	< 0.001	0.836
CMAP → CMAP3	0.937	0.031	30.097	< 0.001	0.808
CMAP → CMAP4	0.884	0.030	29.917	< 0.001	0.805
Gender → LO	0.052	0.048	1.083	0.279	0.037
Age → LO	−0.028	0.021	−1.333	0.182	−0.052
Marital status → LO	0.067	0.059	1.136	0.256	0.039
Education → LO	−0.019	0.030	−0.633	0.527	−0.021
Employment status → LO	0.071	0.055	1.291	0.197	0.041
Housing arrangement → LO	0.083	0.064	1.297	0.195	0.045
Frequency → LO	−0.079	0.046	−1.717	0.086	−0.064

To further examine the robustness of the associations with loneliness, demographic and background variables were included as covariates in the loneliness equation. Specifically, gender, age, marital status, education level, employment status, housing arrangement, and frequency of community music activity participation were entered as covariates predicting loneliness. After accounting for these variables, the associations of community music activity participation, perceived restorativeness, and meaning in life with loneliness remained significant. None of the covariates showed a significant association with loneliness in the current model (all *p* > 0.05), with standardized coefficients ranging from −0.064 to 0.045. These results suggest that the associations of CMAP, PR, and ML with loneliness were not solely attributable to these background characteristics.

Standardized direct and indirect effects were further examined using bias-corrected bootstrap resampling with 5,000 iterations. The bootstrap procedure was used to obtain 95% confidence intervals and bootstrap-based *p* values for the standardized effects. Because the *p* values reported in this section were derived from bootstrap resampling, they may differ from the maximum-likelihood-based *p* values reported for the structural path coefficients reported in Table 3.

See Table 4, the standardized total indirect effect, calculated as the sum of the three indirect pathways (CMAP → PR → LO, CMAP → ML → LO, and CMAP → PR → ML → LO), was −0.184, with a 95% bias-corrected bootstrap confidence interval of [−0.276, −0.102] and a bootstrap *p* value of less than 0.001. The indirect pathways collectively accounted for approximately 47.9% of the standardized total association.

**Table 4:** Bootstrap estimates of standardized direct and indirect associations.

Parameter	Standardized effect estimate	SE	Lower	Upper	*p*
CMAP →PR → ML → LO Chain Ind	−0.023	0.011	−0.051	−0.005	0.010
CMAP →PR → LO → Ind	−0.066	0.023	−0.111	−0.023	0.004
CMAP →ML → LO → Ind	−0.095	0.043	−0.195	−0.018	0.014
CMAP →LO → Direct	−0.200	0.063	−0.316	−0.064	0.004
CMAP →LO → Total	−0.384	0.034	−0.448	−0.32	0.001

All effect estimates reported in this table are standardized. Confidence intervals and p values were obtained using bias-corrected bootstrap resampling with 5,000 iterations. The *p* values may differ from the maximum-likelihood-based *p* values reported in Table 3 because they were obtained using different inferential procedures.

The standardized direct effect of CMAP on loneliness remained significant in the bootstrap analysis [estimate = −0.200, 95% bias-corrected CI (−0.316, −0.064), bootstrap *p* = 0.004]. The standardized total effect was also significant [estimate = −0.384, 95% bias-corrected CI (−0.448, −0.320), bootstrap *p* = 0.001]. The pattern of results is statistically consistent with partial indirect effects, with both the direct and indirect pathways remaining significant. However, given the cross-sectional nature of the data, these findings should be interpreted as reflecting indirect associations rather than causal sequencing.

### 4.5 Sensitivity analysis

Several sensitivity analyses were conducted to examine whether the main findings were robust to alternative construct specifications and structural assumptions.

First, to examine whether the structural findings depended on modeling meaning in life (ML) as a higher-order construct, a sensitivity analysis was conducted in which presence of meaning (PM) and search for meaning (SM) were modeled as separate first-order latent variables. The directions of the principal structural associations remained consistent with those observed in the primary model. However, after partitioning the shared variance between the two meaning dimensions, their unique associations with loneliness were attenuated. Specifically, the path from PM to loneliness was marginally significant (*β* = −0.077, *p* = 0.064), whereas the path from SM to loneliness was not significant (*β* = −0.062, *p* = 0.211). Similarly, the dimension-specific serial indirect effects were weaker (CMAP → PR → PM → LO: *p* = 0.051; CMAP → PR → SM → LO: *p* = 0.241), although the combined indirect effect remained significant [estimate = −0.036, BC 95% CI (−0.072, −0.001), *p* = 0.048]. These findings suggest that the indirect association involving meaning in life was not attributable exclusively to either meaning dimension but rather reflected variance shared across the broader meaning-in-life construct. Full results are reported in Appendix Tables A3, A4.

Second, to evaluate whether the findings were influenced by the operationalization of loneliness, the structural model was re-estimated with loneliness specified as a latent construct indicated by the six ULS-6 items rather than as an observed composite score. The six indicators showed strong factor loadings (standardized loadings = 0.789–0.825, all *p* < 0.001; CR = 0.920, AVE = 0.656). The principal structural associations remained substantively unchanged, including CMAP → PR (*β* = 0.407), CMAP → ML (*β* = 0.503), PR → ML (*β* = 0.293), and ML → LO (*β* = −0.189). The serial indirect association CMAP → PR → ML → LO was also highly comparable to that observed in the primary model [estimate = −0.023, BC 95% CI (−0.050, −0.006)]. These results indicate that the main findings were robust to an alternative latent-variable specification of loneliness. Full results are presented in Appendix Table A5.

Third, alternative structural models were estimated to examine whether the proposed ordering of variables could be distinguished from theoretically plausible alternatives. The model reversing the ordering of perceived restorativeness and meaning in life (CMAP → ML → PR → LO) and the reverse-direction model (LO → ML → PR → CMAP) showed identical fit indices, AIC, and BIC values compared with the proposed model. Therefore, the cross-sectional data did not allow statistical discrimination among these directional specifications. The proposed CMAP → PR → ML → LO sequence was retained as a theoretically informed model based on the conceptual framework of the study rather than as evidence of temporal or causal ordering. Detailed model comparisons are reported in Appendix Table A6.

## 5 Discussion

### 5.1 Association between community music participation and loneliness

The findings showed that, among young adults living alone who already had experience of community music participation, higher levels of community music activity participation were associated with lower levels of loneliness. The standardized direct association remained negative after perceived restorativeness, meaning in life, and background covariates were taken into account (β = −0.200), while the standardized total association was −0.384. These effect estimates suggest that variation in participation was meaningfully, although not strongly, associated with loneliness within this participant sample, and that the proposed indirect pathways do not fully account for the overall association. Importantly, because the study did not include non-participants, these findings should not be interpreted as showing that community music participants experience lower loneliness than individuals who do not participate in community music activities.

One possible interpretation of this association concerns the socially embedded and participatory qualities of community music activities. For young adults living alone, participation in activities such as collective singing, instrumental performance, rehearsals, and other forms of collaborative music-making may provide repeated opportunities for shared presence, interpersonal interaction, coordinated participation, and emotional expression. These experiences may be particularly relevant for individuals whose everyday lives involve limited stable companionship or relatively weak opportunities for sustained social interaction. In this sense, the association between community music participation and loneliness may relate not only to engagement with music itself, but also to the relational and participatory experiences embedded within the activity context.

Shared musical participation may also provide opportunities for emotional resonance and a sense of involvement in collective activity. Through repeated interaction, shared attention, and coordinated engagement, participants may experience stronger perceptions of social connectedness and community involvement, which are conceptually relevant to loneliness as a subjective experience of insufficient or unsatisfactory social connection. Accordingly, the present findings are consistent with the possibility that community music participation represents a socially meaningful context in which young adults living alone can experience interpersonal engagement and emotional connection. Given the cross-sectional design, this relationship should be interpreted as concurrent rather than causal.

### 5.2 Serial indirect association involving perceived restorativeness and meaning in life

The direct and indirect association analysis identified indirect associations between community music activity participation and loneliness involving perceived restorativeness and meaning in life. The standardized total indirect association was −0.184, while the standardized direct association remained −0.200 and the standardized total association was −0.384. Among the specific indirect associations, the serial association involving both perceived restorativeness and meaning in life was relatively modest (−0.023) compared with the associations involving perceived restorativeness alone (−0.066) and meaning in life alone (−0.095). These estimates indicate that the proposed serial pathway represents a meaningful but limited component of the overall association rather than a dominant or comprehensive explanation.

The observed pattern is theoretically consistent with the possibility that restorative and meaning-related evaluations are jointly relevant to the relationship between community music participation and loneliness. Higher participation was associated with stronger perceived restorativeness, perceived restorativeness was associated with greater meaning in life, and meaning in life was associated with lower loneliness. Perceived restorativeness may reflect participants’ evaluations of community music as a psychologically supportive and engaging experiential context, while meaning in life may capture broader evaluations of purpose, value, and personal significance. Self-Determination Theory provides a complementary interpretive perspective for these meaning-related experiences, although basic psychological need satisfaction was not directly measured in the present study.

The relatively small serial indirect association also indicates that perceived restorativeness and meaning in life account for only part of the broader pattern linking community music participation with loneliness. Other factors not examined in the present model, including social support, belongingness, relationship quality, personality, and broader community engagement, may also be relevant. Because the data were collected cross-sectionally, the serial ordering represented in the model should be regarded as theoretically specified rather than temporally established.

### 5.3 Comparison with previous studies and contextual interpretation

The present findings are broadly consistent with previous research linking community and group music participation with social connectedness, belongingness, emotional well-being, and lower loneliness. Previous studies have suggested that shared musical engagement may facilitate social integration and positive emotional experiences through interpersonal interaction, collective participation, and sustained involvement. For example, Gomes et al. (17) reported associations between group singing and stronger social integration and lower loneliness among older adults, while Martín et al. (61) highlighted the relevance of music-related activities to emotional regulation. Cao et al. (62) similarly reported associations between music participation, psychological wellbeing, and loneliness among young adults, and O’Rourke et al. (63) emphasized the immersive and socially engaging qualities of group musical participation. The present study adds to this literature by examining perceived restorativeness and meaning in life as additional psychological correlates within the relationship between community music participation and loneliness among young adults living alone.

At the same time, the present findings should not be interpreted as evidence that the observed restorative, relational, or meaning-related experiences are unique to music. Community music activities combine musical engagement with broader characteristics of collective participation, including shared presence, coordinated activity, interpersonal interaction, emotional involvement, and opportunities for self-expression. Similar experiential qualities may also occur in other socially engaging community activities, such as group exercise, volunteering, community arts, sports, or other forms of collective participation. Because the present study did not include an active non-musical comparison group, it cannot isolate the contribution of musical engagement from these more general participatory characteristics. The findings therefore demonstrate associations within the specific context of community music participation rather than a unique advantage of music over other forms of socially meaningful community engagement.

The geographical setting is also relevant to the interpretation of these findings. Participants were recruited from Beijing, Shanghai, and Shenzhen, three highly urbanized first-tier cities characterized by substantial population mobility, high levels of independent living, and comparatively extensive community and cultural resources. These urban conditions may be relevant in two ways. On the one hand, residential mobility, work- and education-related relocation, and relatively unstable everyday companionship may make opportunities for repeated social connection particularly salient for some young adults living alone. On the other hand, comparatively developed cultural infrastructure and organized community activities may provide greater access to structured forms of collective participation, including community music. Community music participation in these cities should therefore be understood within a specific urban social ecology in which mobility, living arrangements, community resources, and opportunities for cultural participation intersect.

Previous research has examined multidimensional community interventions, social-support-based approaches, and technology-mediated forms of social connectedness (64, 65), while the effectiveness of loneliness interventions has been shown to vary across populations and settings (66). Within this broader literature, community music is best understood as one culturally embedded and socially engaging form of community participation rather than as an established loneliness-reduction intervention. Whether similar associations occur across other forms of community participation, or in smaller cities and rural settings with different social and cultural infrastructures, remains an important question for future comparative research.

### 5.4 Theoretical contributions: perceived restorativeness in community music participation

One of the main theoretical contributions of the present study lies in considering perceived restorativeness within an active and socially embedded form of cultural participation. Although Attention Restoration Theory (ART) was originally developed primarily in relation to physical and natural environments, subsequent research has suggested that restorative qualities may also be perceived through auditory experience. Payne developed the Perceived Restorativeness Soundscape Scale to assess the perceived restorative potential of soundscapes, providing an important basis for considering restoration beyond predominantly visual environmental settings (67). This line of research suggests that auditory environments can be evaluated in terms of experiential qualities conceptually related to psychological restoration.

Research involving music provides a further bridge to the present study. Ho and Au (68) found that the presence of street performance was associated with more restorative evaluations of public space, suggesting that musical and performative elements may contribute to how environmental experience is perceived. Complementary experimental evidence has also indicated that music may be relevant to the recovery of attentional resources. Baldwin and Lewis (69) reported that positively valenced music, particularly slow-tempo positive music, was associated with improved sustained-attention performance following attentional fatigue. Taken together, these findings suggest that auditory and musical experiences may be relevant to both perceived restoration and attentional recovery. However, much of the existing evidence concerns soundscape perception, musical exposure, audience experience, or individual listening. Active and socially interactive forms of collective music participation have received comparatively less attention from the perspective of perceived restorativeness.

The present study contributes to this discussion by examining perceived restorativeness in the context of active community music participation. Community music activities involve more than exposure to auditory stimuli. Collective singing, instrumental performance, rehearsal, and collaborative music-making combine musical engagement with shared attention, coordinated action, interpersonal interaction, emotional involvement, and sensory immersion. These experiential characteristics may correspond to the restorative dimensions described by ART. Temporary psychological distance from everyday pressures may be experienced as being away; rhythm, melody, and immersive collective engagement may support fascination; the continuity and internal structure of rehearsal and collaborative performance may contribute to extent; and a perceived fit between participants’ needs, modes of participation, and the social–emotional atmosphere of the activity may be reflected in compatibility. From this perspective, community music participation may be understood as a psychologically and socially constructed experiential context in which participants perceive restorative qualities, rather than simply as exposure to a pleasant auditory environment.

This interpretation nevertheless requires clear theoretical boundaries. Because the Perceived Restorativeness Scale was contextually adapted specifically to community music participation, the present findings should be understood as preliminary evidence that participants evaluate these activity experiences in ways that are consistent with ART-related restorative dimensions. They should not be interpreted as independent empirical validation that ART has been definitively extended from physical or natural environments to active community music participation. In addition, the present study did not include comparison groups engaged in non-musical community activities. It therefore cannot determine whether the observed restorative experiences arise specifically from musical engagement or from broader characteristics of socially meaningful collective participation, such as shared attention, coordinated activity, interpersonal connection, emotional involvement, and opportunities for self-expression. Similar experiential qualities may also occur in group exercise, volunteering, community arts, sports, or other forms of socially engaging community participation. Accordingly, the theoretical contribution of the present study is not to establish a unique restorative effect of music, but to identify perceived restorativeness as a potentially useful conceptual framework for examining active, socially immersive, and culturally embedded participatory contexts, with community music representing one specific context in which this possibility can be explored.

### 5.5 Practical implications

From a practical perspective, the present findings suggest that community music may represent one promising form of socially engaging community participation for young adults living alone who are already willing or able to participate in such activities. Within this participant group, variation in community music participation was associated with loneliness, perceived restorativeness, and meaning in life. Programs involving collective singing, collaborative music-making, or interactive music workshops may provide opportunities for interpersonal connection, emotional expression, restorative experience, and meaningful engagement. Their potential value may lie not only in musical content itself, but also in opportunities for shared attention, coordinated participation, repeated social interaction, and emotionally meaningful involvement (70, 71).

However, these findings should not be interpreted as evidence that introducing community music programs will necessarily reduce loneliness or that people who participate in community music experience lower loneliness than non-participants. All respondents in the present study had prior community music participation, and individuals who voluntarily engage in such activities may differ systematically from non-participants in social motivation, openness, cultural engagement, existing social networks, psychological well-being, or access to community resources. Moreover, because the study did not directly compare community music with non-musical forms of community participation, the findings do not demonstrate that music-based activities are more effective than other socially engaging collective activities. Community music should therefore be regarded as a potentially useful context for future intervention development and evaluation rather than as an established loneliness-reduction intervention. Longitudinal and controlled comparative studies involving both non-participating and active non-musical comparison groups are needed before stronger practical conclusions can be drawn.

### 5.6 Limitations and future research

Several limitations define the boundaries of interpretation. First, the cross-sectional design does not establish causal direction or temporal ordering among community music participation, perceived restorativeness, meaning in life, and loneliness. The observed indirect associations are consistent with the proposed theoretical model but do not demonstrate a temporal process, and reverse or reciprocal relationships remain possible. Longitudinal and controlled studies are therefore needed to examine temporal ordering and change over time.

Second, the recruitment strategy and geographical scope limit representativeness. All respondents had prior community music participation and were recruited primarily through related activity settings, so the study cannot determine whether participants experience lower loneliness than non-participants. Self-selection may also reflect differences in sociability, openness, cultural engagement, social networks, psychological well-being, or access to community resources. In addition, participants were drawn from Beijing, Shanghai, and Shenzhen, and no active non-musical comparison group was included. The findings therefore apply most directly to variation among existing participants and cannot establish effects unique to music or generalize readily to other regions.

Third, community music participation and living-alone experiences were heterogeneous. Although CMAP captured multiple aspects of engagement rather than participation frequency alone, activity types were not modeled separately, and characteristics such as duration, continuity, intensity, artistic responsibility, and musical expertise were not comprehensively assessed. Potentially relevant confounders, including socioeconomic status, social network size, duration and voluntariness of living alone, musical background, prior mental-health status, and personality, were also not fully measured or controlled. Future research should use more differentiated measures of participation, exposure, living arrangements, and relevant background characteristics.

Finally, several measurement and model-specification issues remain. All principal constructs were assessed by self-report within the same survey, creating potential shared method variance. The contextually adapted Perceived Restorativeness Scale also cannot distinguish restorative perceptions arising from the venue, musical activity, or interpersonal experience. In addition, meaning in life was modeled as a broader construct comprising presence and search for meaning, while loneliness was represented by the observed ULS-6 composite score. Although sensitivity and robustness analyses examined alternative specifications of meaning in life, loneliness, and structural ordering, these analyses do not eliminate the limitations associated with self-report measurement, model specification, or the cross-sectional design. Future research should continue to evaluate the stability of the findings using multimethod, longitudinal, and alternative modeling approaches. Future studies should incorporate multimethod measurement and examine alternative measurement and structural specifications.

## 6 Conclusion

This study examined the associations among community music activity participation, perceived restorativeness, meaning in life, and loneliness among young adults living alone with prior community music participation in China. Higher levels of community music participation were associated with lower loneliness, and perceived restorativeness and meaning in life were involved in both separate and serial indirect associations. However, the serial indirect association was relatively modest, while the direct association remained evident, indicating that the proposed psychological pathway represents only a partial rather than comprehensive explanation of the relationship between community music participation and loneliness.

The findings also suggest that perceived restorativeness may provide a useful conceptual framework for understanding active, socially immersive, and culturally embedded forms of participation. In the present study, community music was experienced in ways consistent with ART-related restorative dimensions, but the findings do not constitute definitive validation of an extension of ART to active music participation. Nor can they establish that the observed restorative and meaning-related experiences are unique to music rather than shared with other forms of socially meaningful community engagement. Given the cross-sectional and observational design, the results should therefore be interpreted as concurrent associations rather than causal or temporal processes. Community music may represent a promising context for future community-based intervention development, but longitudinal and controlled comparative studies are needed before stronger causal, practical, or music-specific conclusions can be drawn.

## Data Availability

The raw data supporting the conclusions of this article will be made available by the authors, without undue reservation.
