# Supplementary material for: Community music activity participation and loneliness among Chinese young adults living alone
Source: Front Public Health. 2026 Sep 3;14:1930459. doi: 10.3389/fpubh.2026.1930459 (PMC13582354; doi:10.3389/fpubh.2026.1930459)
Supplement: Supplementary file 1 [file Supplementary_file_1.docx]

**Appendix A**

**Table A1. Results of Confirmatory Factor Analysis**

| Latent Variable | Measurement Item | Standardized Loading Coefficient | AVE | CR |
| --- | --- | --- | --- | --- |
| CMAP | CMAP1 | 0.858 | 0.684 | 0.896 |
|  | CMAP2 | 0.836 |  |  |
|  | CMAP3 | 0.808 |  |  |
|  | CMAP4 | 0.805 |  |  |
| BE | BE1 | 0.792 | 0.637 | 0.840 |
|  | BE2 | 0.817 |  |  |
|  | BE3 | 0.785 |  |  |
| EX | EX1 | 0.818 | 0.669 | 0.859 |
|  | EX2 | 0.833 |  |  |
|  | EX3 | 0.803 |  |  |
| FA | FA1 | 0.862 | 0.731 | 0.891 |
|  | FA2 | 0.840 |  |  |
|  | FA3 | 0.862 |  |  |
| CO | CO1 | 0.905 | 0.768 | 0.869 |
|  | CO2 | 0.847 |  |  |
| PM | PM1 | 0.815 | 0.648 | 0.902 |
|  | PM2 | 0.810 |  |  |
|  | PM3 | 0.788 |  |  |
|  | PM4 | 0.787 |  |  |
|  | PM5 | 0.825 |  |  |
| SM | SM1 | 0.724 | 0.569 | 0.841 |
|  | SM2 | 0.760 |  |  |
|  | SM3 | 0.769 |  |  |
|  | SM4 | 0.764 |  |  |
| LO | LO1 | 0.790 | 0.656 | 0.920 |
|  | LO2 | 0.798 |  |  |
|  | LO3 | 0.825 |  |  |
|  | LO4 | 0.824 |  |  |
|  | LO5 | 0.821 |  |  |
|  | LO6 | 0.802 |  |  |

**Table A2. Correlations Among Latent Variables and Square Roots of AVE**

| Latent Variable | **CMAP** | **BE** | **EX** | **FA** | **CO** | **PM** | **SM** | **LO** |
| --- | --- | --- | --- | --- | --- | --- | --- | --- |
| **CMAP** | ****0.827**** |  |  |  |  |  |  |  |
| **BE** | 0.277 | ****0.798**** |  |  |  |  |  |  |
| **EX** | 0.316 | 0.515 | ****0.818**** |  |  |  |  |  |
| **FA** | 0.259 | 0.547 | 0.536 | ****0.855**** |  |  |  |  |
| **CO** | 0.310 | 0.489 | 0.500 | 0.408 | ****0.876**** |  |  |  |
| **PM** | 0.382 | 0.208 | 0.248 | 0.190 | 0.201 | ****0.805**** |  |  |
| **SM** | 0.496 | 0.294 | 0.295 | 0.293 | 0.245 | 0.491 | ****0.754**** |  |
| **LO** | -0.384 | -0.230 | -0.241 | -0.282 | -0.200 | -0.263 | -0.302 | ****0.810**** |

*Note: Bolded diagonal values are the square roots of AVE; off-diagonal values are latent variable correlations. Discriminant validity is supported if each bolded value exceeds the corresponding correlations in its row and column.*

**Table A3. Sensitivity Analysis: Path Coefficients with PM and SM as Separate Mediators**

| **Path** | **β (Std.)** | **S.E.** | **z** | **p** |
| --- | --- | --- | --- | --- |
| PM ← CMAP | 0.316 | 0.040 | 7.971 | < .001 |
| PM ← PR | 0.195 | 0.048 | 4.100 | < .001 |
| SM ← CMAP | 0.402 | 0.042 | 9.483 | < .001 |
| SM ← PR | 0.254 | 0.052 | 4.896 | < .001 |
| LO ← CMAP | -0.247 | 0.047 | -5.226 | < .001 |
| LO ← PR | -0.190 | 0.048 | -3.928 | < .001 |
| LO ← PM | -0.077 | 0.042 | -1.852 | 0.064 |
| LO ← SM | -0.062 | 0.050 | -1.250 | 0.211 |

**Table A4. Sensitivity Analysis: Bootstrap Estimates of Direct and Indirect Associations with PM and SM as Separate Mediators**

| **Indirect effect** | **Est.** | **BC 95% CI** | **p** |
| --- | --- | --- | --- |
| Total indirect | -0.036 | [-0.072, -0.001] | 0.048 |
| CMAP→PR→PM→LO | -0.018 | [-0.039, 0.000] | 0.051 |
| CMAP→PR→SM→LO | -0.018 | [-0.046, 0.012] | 0.241 |
| Direct | -0.181 | [-0.249, -0.112] | < .001 |

**Table A5. Robustness Analysis Comparing Composite-Score and Latent-Variable Specifications of Loneliness**

| **Parameter** | **Primary model: Composite LO** | **Robustness model: Latent LO** |
| --- | --- | --- |
| CMAP → PR, β | 0.407 | 0.407 |
| CMAP → ML, β | 0.503 | 0.503 |
| PR → ML, β | 0.293 | 0.293 |
| ML → LO, β | −0.189 | −0.189 |
| CMAP → PR → ML → LO, indirect effect | −0.023 | −0.023 |
| BC 95% CI for serial indirect effect | [−0.051, −0.005] | [−0.050, −0.006] |

Table A6. Comparison of Alternative Structural Models

| **Model** | **χ²** | **df** | **CFI** | **TLI** | **RMSEA (90% CI)** | **SRMR** | **AIC** | **BIC** |
| --- | --- | --- | --- | --- | --- | --- | --- | --- |
| A: CMAP → PR → ML → LO | 877.944 | 393 | 0.973 | 0.970 | 0.035 (0.032, 0.039) | 0.029 | 80783.373 | 81282.008 |
| B: CMAP → ML → PR → LO | 877.944 | 393 | 0.973 | 0.970 | 0.035 (0.032, 0.039) | 0.029 | 80783.373 | 81282.008 |
| C: LO → ML → PR → CMAP | 877.944 | 393 | 0.973 | 0.970 | 0.035 (0.032, 0.039) | 0.029 | 80783.373 | 81282.008 |

*Note. The three models were statistically equivalent and therefore could not be distinguished on the basis of global fit indices.*
